# Epistemic Rationality Begins Unreflectively

**DOI:** 10.1007/s10670-025-00977-x

**Published:** 2025-06-13

**Authors:** Giacomo Melis, Kirsten H. Blakey

**Affiliations:** 1 Division of Law and Philosophy, University of Stirling, Stirling, Scotland UK; 2 Division of Psychology, University of Stirling, Stirling, Scotland UK; 3 Psychology, University of Toronto, Toronto, ON Canada

## Abstract

Recent research in analytic epistemology suggests that one can form a rational belief without being in the position to identify and assess the evidence in its support. The reach of such unreflective responses to evidence has been explored in internalist and externalist theories of epistemic justification. It is also at work in defences of the rationality of non-human animals and young children. Unreflective responsiveness to evidence is in tension with reflective accounts, according to which being in the position to identify and assess the relevant reasons is necessary for rational belief and action. We investigate the relation between these two rival characterizations of rational belief by engaging with recent work in developmental psychology and integrating it with lessons coming from the study of epistemic defeaters. We contend that the roots of epistemic rationality lie in what subjects do at the unreflective level. The arguments we provide speak against the widely held notion that the reflective minds of adult humans differ in kind from the presumed unreflective minds of young children and non-human animals.

## Introduction

From the infinite regresses outlined by Boghossian (2003), Kornblith (2012) and Wright (2012), to Williamson’s (2000) anti-luminosity argument, recent analytic epistemology is critical of the idea that the justification of an evidence-based belief always requires one to be in the position to evaluate one’s evidence. Rather, there must be cases where subjects form beliefs rationally without having reflective access to the evidence supporting those beliefs. Competing theories of evidence make room for such cases. For example, both mentalist internalism—according to which one’s evidence is determined by a subset of one’s non-factive mental states (e.g., Conee & Feldman, 2004; Wedgwood, 2017)—and evidential externalism—which characterizes evidence in terms of agent-independent facts (e.g.,Lord, 2018; Williamson, 2000)—allow that one can be justified in believing *p* without being in the position to identify and assess the evidence in favour of doing so. Moreover, the broader notion of unreflective responsiveness to (practical or epistemic) reasons is at work in defences of the rationality of non-human animals and young children (e.g., Danón & Kalpokas, 2023; Dretske, 2006; Glock, 2019; Hurley, 2006; Mercier & Sperber, 2018).

However, many philosophers still maintain that unreflective responsiveness to reasons or evidence, on its own, is not sufficient for rational belief (e.g., Brandom, 2010; Marcus, 2021; McDowell, 1994; Moran, 2001). These philosophers typically admit that human adults often form beliefs unreflectively and yet insist that such beliefs are apt to be justified or rational only in so far as the believing subject can, ex-post, ask herself whether she has good reasons to hold them. In the reflectivist picture, the capacity to raise and answer such question is taken to be necessary for (i) identifying, and (ii) assessing one’s evidence or reasons, which in turn are taken to be the distinctive features of rational agency (e.g.,Boyle, 2018; Korsgaard, 2018). Since replying to a request for reasons in support of one’s belief, in effect, requires one to formulate a thought like < I believe that *p* because of *q* > , or < *q* is a good reason to believe that *p* > , in the reflectivist framework responding to evidence or reasons requires the metacognitive capacity to formulate thoughts about other thoughts. It is a consequence of the view that the beliefs of presumed unreflective subjects like very young children and non-human animals can be rational only in a loose or metaphorical sense.

The relation between these two competing characterizations of what it takes to respond to evidence or reasons remains unexplored. It is an open question whether the two notions are a sign of dualism about evidence-responsiveness, or instead they share some unifying feature that makes them two manifestations of the same capacity. This issue is of central importance to understand the nature of rational agency. The dualist position may vindicate a special status of reflective subjects with respect to rationality, while an underlying unity would see unreflective and reflective subjects as both capable of engaging in rational endeavours—if at different levels of proficiency. Indeed, this is one way to reconstruct the opposition between philosophers who claim that rationality is uniquely human (e.g., Boyle, 2018; Davidson, 1982; Korsgaard, 2018; McDowell, 1994) and those who extend it to non-human animals (e.g., Danón & Kalpokas, 2023; Dretske, 2006; Glock, 2019; Hurley, 2006).

Since human children make the transition from a state in which they are only capable of unreflective responses to a state where they can also engage in reflective scrutiny of reasons, addressing the issue invites taking a developmental perspective. How do children achieve the transition? More specifically, does the transition mark a *transformation* in children’s cognitive abilities which turns non-rational subjects into rational ones, or are the cognitive abilities at work in children’s early unreflective belief-revision essentially the same as those underwriting their later reflective assessments?

We argue that recent work in contemporary epistemology and developmental psychology suggests that there is a cognitive mechanism that explains the transition from unreflective to reflective responsiveness to evidence, and which speaks against dualism. Such a cognitive mechanism is the acquisition and response to some undermining defeaters. In the view that we will illustrate, the abilities at work in unreflective evidence-based belief-formation of very young children are the very abilities that ground adults’ reflective responses. If so, rational agency is not the upshot of the acquisition of reflective skills, but it is already at work in the cognitive achievements of unreflective subjects.

The plan of the paper is the following. In § 2 we explain the relevant notion of reflection and further articulate the opposition between the two views outlined. In § 3 we present the prima facie argument in defence of the continuity between unreflective and reflective responses to evidence. In §§ 4 and 5 we refine the original argument by addressing two objections in turn. In § 6 we draw some final considerations.

## Reflective vs Unreflective Responses to Evidence

The term “reflection” is often used as a near synonymous of “reasoning” or “thinking,” especially when one wants to indicate that the relevant reasoning is executed with care and attention. Among the many things people can reflect on are the reasons or evidence that speak in favour or against adopting specific doxastic attitudes. In this paper, we use the term “reflection” narrowly, as short for “thinking about reasons or evidence.” Similarly, we use cognate expressions such as “reflectivism” and “reflectivist” to refer to the view that being in the position to scrutinize one’s reasons or evidence is necessary for rational belief.

To appreciate the relevant notion of reflection, it is important to keep in mind that in the present debate rationality is characterized as the capacity to respond to evidence or reasons. To say that an epistemically rational subject is a subject who can respond to evidence is to say that such subject can form beliefs in the light of an appreciation of the normative force of the relevant evidence—that, in ways to be spelled out, the subject appreciates the relation of evidential support between the evidence and a doxastic attitude. Thus, in the present context rationality is decidedly *not* cashed out in purely formal (e.g., conformity with the axioms of probability calculus), instrumentalist (e.g., efficiency in reaching a goal or in executing means-end calculations), or structural (e.g., attitudinal coherence à la Broome, 2013, or Scanlon, 1998) terms. Rather, it is the so-called “substantive” rationality, as discussed by, e.g., Kiesewetter (2017) and Lord (2018). With this background, appeals to reflection as what makes subjects rational are claims about *the content* of reflective thoughts. While details may differ in specific versions of the reflectivist view, the two features already mentioned in § 1 provide a minimal characterization that will serve our purposes. To wit: A reflectively justified belief is based on, or can be ex-post sustained by, (i) the conscious identification, and (ii) the conscious evaluation of the relevant evidence.^1^ By contrast, an unreflectively justified belief is *not* based on, and *cannot* be sustained ex-post by, the conscious identification and evaluation of the relevant evidence. Reflectively justified beliefs, but not unreflectively justified ones, require that the subject is in the position to vouch for the justification of the relevant belief.^2^ To vouch for the justification of one’s belief, one has to possess some epistemic concepts in order to be able to form thoughts like < the evidence suggests that P is true > , or < a reliable source suggests that P > . Thus, reflective responses to evidence require grasp of epistemic concepts. We’ll discuss the role of epistemic concepts in reflective thought in the following sections. For now, the point is just that the distinction between reflective and unreflective beliefs that matters here is different from similar distinctions drawn in terms of cognitive processing, which are popular in some debates in cognitive psychology and in the philosophy of cognitive science and which tend to use the term “reflection” in a sense that is closer to the common notion of “careful reasoning”.

Examples of the latter characterizations are the Dual Process Theory defended by, e.g., Evans and Stanovich (2013) and the Dual Factor theory advocated by, e.g., Byrd (2021, 2022). The Dual Process Theory distinguishes *intuitive* judgments resulting from fast, relatively undemanding and automatic processes from *reflective* judgments which are due to slow, high-effort and controlled processes. Simplifying, what is distinctive of reflective thought in this picture is the role played by working memory.^3^ Similarly, the Dual Factor Theory sees the distinctive feature of reflective thought in “stepping back and consciously reconsidering” (Byrd, 2021, p. 3) with the goal of double-checking one’s initial judgments. Both theories advocate gradualist approaches between unreflective and reflective thinking, which are broadly congenial to the position we will defend in the rest of the paper (cf. footnote 20 below). Yet, the characterizations of the reflective/unreflective distinction that they rely on differ from the one that is relevant here in that they concern primarily the level of cognitive processing rather than the content of thoughts.

One way to appreciate this difference is to consider so called “cognitive reflection tests,” inspired by Kahneman and Frederick (2002) and often used to measure reflection in cognitive psychology. These tests typically consist in a series of little puzzles such as “If you were running a race, and you passed the person in 2nd place, what place would you be in now?” Now, for most subjects unused to engage with similar questions, the lured, automatic reply is the erroneous “1st place”. Hence, most subjects have to resist the pull of the lured judgment and think a little harder in order to get to the correct answer: “2nd place”. Because of this, success in this test is taken to be evidence of reflective thinking.^4^ Let’s suppose that formulating the right answer requires one to think that, say, whoever is running behind the 2nd in the race is in 3rd position, and if the 3rd passes the runner in front, they will become 2nd. This would surely be a case of *inferential* reasoning, which would contrast with the immediate reaction of subjects who reply “1st place” right away. Yet, it would not thereby involve the deployment of epistemic concepts as required by the notion of reflective responsiveness to reasons that is relevant here. One can go through the reasoning just rehearsed without ever wondering whether the evidence she possesses supports “2nd place” or “1st place”. More generally, one can perform successfully sophisticated pieces of inferential reasoning without considering whether the premises really support the conclusion.^5^ But that is what reflective responsiveness to reasons would, in the circumstances, require. The project of understanding the role of reflection—understood as effortful or controlled inferential reasoning—in human adults is orthogonal to the question of how presumed unreflective subjects such as young children may begin to engage in reflective thought—understood in terms of reflective responsiveness to reasons. We invite readers invested in the psychological literature mentioned above, which tends to use “reflection” and cognates as “conscious effortful reasoning,” to keep in mind our narrow use of “reflection” as short for “reflection on reasons.”

Having clarified the relevant notion of reflection, let’s expand on the contrast between unreflective and reflective responsiveness to reasons. The difference between the two characterizations concerns how the notion of appreciating the normative force of reasons is unpacked. Reflectivists maintain that it requires *thinking of reasons as reasons*; their opponents contend that it may be manifested through mere *sensitivity to reasons*. As already noted, the issue is crucial to understand rational agency and the relation between reflective and unreflective minds. If rational agency requires thinking of reasons as reasons, unreflective subjects would not be, properly speaking, rational. Unreflective minds would be unable to appreciate the normative force of reasons and would thereby be blind as to why they believe the things they believe and do the things they do. Reflectivists needn’t deny that unreflective subjects act intelligently in various ways but insist that, without the capacity to appreciate why the things they believe and do are the right (or wrong!) things to believe and do, unreflective subjects lack the self-understanding needed to count as genuine rational subjects. Reflectivists are committed to saying that reflective and unreflective minds are of a different kind. That is to say, even the capacities for movement and perception are supposed to take an altogether different form in reflective subjects. For example, McDowell (1994) famously argued that, in reflective but not in unreflective subjects, perceptual contents are conceptual.^6^ In the reflectivist picture, reflective abilities *transform* the minds of subjects who possess them and make them altogether different from unreflective minds: Reflective subjects are supposedly no longer capable of *mere* unreflective responses to reasons.^7^ Thus, the reflectivist approach endorses a dualism between unreflective and reflective minds. Minimally, the relevant dualism is what Bar-On (2009, 2018) dubs “mind-mind dualism” to mark the distinction with the more familiar mind–body dualism of the Cartesian tradition: The latter, but not the former, is committed to the existence of immaterial substances.^8^

Against the reflectivist picture, philosophers who allow for unreflective responses to evidence needn’t postulate any difference in kind between unreflective and reflective minds and can welcome continuity views. The anti-reflectivist approach may be articulated in various ways, and some of them are especially critical of the significance of reflection for rational belief-revision in a wide range of circumstances (e.g., Kornblith, 2012, 2022, Chapter 4). But simply accepting the existence of unreflective responses to evidence, in and of itself, brings no commitment to devaluing the contribution of reflection in the belief-revision of adult humans.

Reflectivists would resist the use of the term “unreflective responsiveness to evidence,” as they deny that there is such a thing. In their picture, forming and revising beliefs—or other mental representations—in the light of changing environment can’t be genuine responsiveness to evidence, unless it is supported by the capacity to think of evidence as evidence. Yet, current epistemology makes room for unreflective responses to evidence; we register this fact and go on to use the related terminology. Nothing hangs on this choice though: The issue of the transition between unreflective and reflective ways of forming and revising one’s representations in the light of changing environments stands regardless of the terms we use to refer to the relevant capacities. We think that the question of the transition is especially important, and it is the question we address. Reflectivists need to come up with an account of that transition that vindicates mind-mind dualism. We propose an account that vindicates mind-mind continuity. If we are right in what is to follow, unreflective responses to evidence constitute the normative and psychological basis of reflective responses and there is no difference in kind between unreflective and reflective minds.^9^

## The Unreflective Basis of Reflective Belief-Revision

Unreflective responses to evidence find their home in first-order belief-formation and revision: Transitions between doxastic attitudes not mediated by thoughts expressing epistemic evaluations. Such transitions involve responses to first-order, but not to higher-order, evidence. Roughly, first-order evidence speaks in favour of taking a doxastic attitude towards a proposition describing some state of affairs, while higher-order evidence speaks in favour of taking an attitude towards the good standing of the first-order evidence. Examples of higher-order evidence are considerations suggesting that one’s evidence is misleading, or that one’s source of evidence is unreliable.^10^ Minimally, first-order belief-revision involves the capacity to respond to ordinary first-order positive evidence—evidence supporting belief in a proposition *p*—and common “overriding” (aka “rebutting”) defeaters—evidence supporting the replacement of belief in *p* with belief in not-*p*.

Adults commonly engage in first-order belief-revision during their daily activities. Suppose that, on the basis of apparent memory, you believe that there are some tomatoes in the pantry but when you get home and check the pantry, you find no tomatoes. On finding no tomatoes you justifiedly replace your original belief with its negation, without having to think about what the evidence is, what it supports, or whether it is strong enough to justify the change in attitude. First-order belief-revision is also part of children’s cognitive repertoire before they are typically considered reflective subjects—that is, before they learn to reply appropriately to requests for reasons in support of their beliefs. A young child may form the belief that the toy is in the red box upon seeing it being placed in the red box but then, once she goes to check the box and sees that it is empty, she automatically replaces her original belief with its negation: < the toy is not in the red box > . Yet, when asked whether and why she’s looking for the toy, or why she believes that the toy is/isn’t in the red box, she may fail to say something relevant, or may not understand the question at all. Indeed, children struggle to justify or explain the reasons for which they form doxastic attitudes until around 5 to 6 years old (Blakey et al., 2021; Butler et al., 2020; Rohwer et al., 2012).^11^

At the unreflective level one forms beliefs in response to (positive or negative) evidence without identifying the evidence as such and without entertaining thoughts about it. As the examples just mentioned illustrate, justified belief in such cases doesn’t require one to think about what the relevant evidence is, what doxastic attitude it supports, or with what strength. Our question is: How can a child who is *only* capable of responding to evidence in the unreflective sense acquire the reflective capacity to identify and assess her evidence?

The study of epistemic defeaters indicates a promising line of inquiry. It is common to distinguish the already mentioned overriders from so-called “undermining” (aka “undercutting”) defeaters (Pollock, 1974, pp. 42–43). While overriders support replacing one’s belief that *p* with the belief that not-*p*, underminers typically suggest that something was wrong in the way one’s belief that *p* was formed. When underminers do so, they constitute (negative) higher-order evidence. For example, learning that the source from which one gathered evidence in support of *p* is unreliable is an undermining defeater for one’s belief that *p*. A rational response to it would be to suspend judgment on *p,* or to reduce one’s credence in *p*. The interesting thing about such a response is that it requires the subject to consciously identify and assess a piece of first-order evidence. To reduce one’s credence or suspend judgment on *p* on the grounds that the evidence in support of *p* came from an unreliable source, one needs to (i) identify the relevant evidence, and (ii) assess it as likely to be misleading.

Hence, responding to the undermining defeaters illustrated is more cognitively demanding than responding to ordinary overriding defeaters. Even more importantly for present purposes, responding to relevant undermining defeaters instantiates the distinctive features of reflective responses to reasons. If so, responding to undermining defeaters like the one described is something that occurs at the reflective level (Melis & Monsó, 2024). In light of this, we can reformulate the original question concerning children’s transition to the reflective level in the following way: How can a young child acquire the capacity to respond to undermining defeaters like the one just described?

If the acquisition of such a capacity can occur without reliance on the metacognitive capacities to formulate thoughts about other thoughts and to reply to requests for reasons, then there is an intermediate stage between unreflective responsiveness to evidence and the full-fledged reflective responses that inspire the reflectivist model outlined in §§ 1–2. Contra dualism, this suggests that unreflective and reflective responsiveness to evidence are not exclusive alternatives, and that there may be continuity between them. By contrast, if the capacity to formulate thoughts about other thoughts or to reply to a request for reasons is needed to acquire the capacity to respond to relevant undermining defeaters, reflective and unreflective responsiveness to reasons may remain insulated from each other. That is to say, by the time children learn to respond to undermining defeaters, the transformation—postulated by the reflectivist approach—of the unreflective, and allegedly non-rational mind, into the reflectively rational mind would have already occurred. Pending an account of that putative transformation, a hypothetical cognitive intertwinement between the emergence of the ability to respond to undermining defeaters and the capacity to formulate thoughts about other thoughts could be part of an argument in defence of dualism.

Let us thus ask how children may acquire the capacity to respond to undermining defeaters like < the source is unreliable > . To ensure that we single out the same cognitive mechanism in children and adults, let’s consider adults first. In many cases, people acquire an undermining defeater like < the source of evidence is unreliable > from a third party. Suppose you’re visiting a new city and ask directions on how to reach the post office from a random person but, as soon as you start walking in the direction indicated by the stranger, a local traffic warden warns you that the person you spoke with is a pathological liar who enjoys misleading people. Through the testimony of the traffic warden, you have thereby *acquired* the undermining defeater, and you *respond* to it by suspending judgment on the location of the post office. In this case, the defeater *destroys*, so to say, your previous justification to believe that the post-office was at a certain location.

In other cases, adults acquire < the source of evidence is unreliable > first-hand. Suppose that you have just joined a new office and are getting to know your new colleagues. After a while you notice that Sam said that he would do things and that he’d be in places—“I’ll be at the meeting”, “I’ll join you folks for lunch”, “I’ll send you that document”…—but, as it turned out, he didn’t do those things and was not in those places. On this basis, you generalize to the conclusion that Sam is not trustworthy. You have thus *acquired* an undermining defeater like < the source is unreliable > . You *respond* to it by suspending judgment on the next declaration of intent Sam makes. In cases like this, the undermining defeater *pre-empts* the formation of future justified beliefs following evidence coming from the source judged to be unreliable. Such cases are especially important in investigating how children may acquire the capacity to respond to undermining defeaters. Their significance may be appreciated by describing the mechanisms underlying the distinct phases of acquisition and response to the defeater.

First, *the acquisition* of < the source is unreliable > is obtained by a generalization over the several not-*p* beliefs that you formed in response to defeaters overriding what Sam said. Those overriding defeaters are constituted by Sam’s absence at the meeting and at lunch, the failure to receive the documents he promised to send, etc. In response to such overriding defeaters, you formed the negation of each of the original beliefs: < Sam is not at the meeting > , < Sam didn’t come for lunch > , and so on. You noticed that all of these overriding defeaters have affected beliefs formed following evidence provided by the same source; namely, Sam. It is this that grounds your generalization to < the source is unreliable > . This is important because it shows that the route that leads you to acquiring the undermining defeater is grounded in first-order thinking. It is constituted by responses to overriding defeaters, and a generalization. These are cognitive mechanisms that are available at the unreflective level.

The second important thing to note is that *to respond* to < the source is unreliable > by suspending judgment on what Sam says next time, you have to: (i) identify what Sam says as a putative piece of evidence, and (ii) assess it as likely to be misleading. As we have seen, you do this because you noticed a pattern such that what seemed like good evidence—what Sam said—often turned out to be not so. The thing to emphasize is that, in identifying a putative piece of evidence and assessing it as likely to be misleading, you ascend to the level of reflective responses to evidence.

On the face of it, the sort of reflective responsiveness to reasons just described is less demanding than, and needn’t rely on, formulating thoughts about other thoughts such as < I [refuse to] believe *p* because of *q* > . There does not seem to be reference to other thoughts or propositions in < the source of evidence is unreliable > . Rather, as noted, its acquisition relied on cognitive capacities available at the unreflective level. If so, a child who can’t yet respond to a verbal request for reasons may be able to do it.

Indeed, recent empirical research may suggest that young children can do generalizations very similar to the one described before they are able to formulate and express thoughts about other thoughts. For example, the seminal study on selective trust conducted by (Koenig et al. 2004) exposed 3- and 4-year-olds to two adults who were asked to name objects with which the children were familiar. One of the adults consistently named the objects correctly while the other consistently named them incorrectly (e.g., calling a “shoe” a ball). When asked whether anyone had said something right/wrong, children in both age groups were able to explicitly identify the misleading informant above chance, suggesting that they had *acquired* an undermining defeater like < the source of evidence is unreliable > . Moreover, a good proportion of these children went on to refuse to accept the name of a novel object (e.g., a woven bamboo object referred to as “toma”) offered by that informant, preferring to endorse the name suggested by the previously reliable informant (e.g., “mido”).^12^ This isn’t enough to conclude that the children in question had *responded* to the undermining defeater, as the experimental set-up leaves room for alternative explanations in terms of unreflective association.^13^ Nevertheless, it suggests we should take seriously the possibility that young children are able to engage in basic forms of reflective responses to reasons. Subsequent studies have found similar patterns of results in domains other than object names (Clément et al., 2004), by removing or postponing the question asking children whether anyone had said something right or wrong (Birch et al., 2008; Corriveau & Harris, 2009), by weakening the degree of reliability/unreliability of the informants (Pasquini et al., 2007), and by running the second phase of the test several days later (Corriveau & Harris, 2009).

Analogous considerations about children’s ability to identify and assess reasons (for action) emerge from recent research on decision making using the marshmallow (delay of gratification) task. In the classic marshmallow task, children have the choice to eat a single marshmallow immediately, or to wait for about 15 min and have two instead. Typically, children give in to temptation after 5–6 min—a result that is commonly explained in terms of children’s still developing capacity for inhibitory control. Yet, other considerations may play a role: After all, waiting makes sense only in a context where children can reasonably expect that a second marshmallow will indeed appear and that the original one won’t be taken away. Kidd et al. (2013) showed that 3- to 5-year-olds who have experienced an experimenter delivering a high value item (e.g., brand new crayons, fancy stickers) after a reasonable short delay (2.5 min) were much more willing to wait for a second marshmallow than children who have experienced an experimenter failing to keep her promise to deliver a high value item after the delay. The former waited 12 min on average, while the latter gave up after 3 min. Michaelson and Munakata (2016) obtained similar results in a set-up where the trustworthiness/untrustworthiness of the experimenter was observed by the child in the experimenter’s interaction with another adult, rather than being experienced first-person. While these studies do include the change of context previously missing,^14^ it may still not be enough to conclude that children judged that specific sources were unreliable. In this case the experimental set-up leaves room for explanations in terms of negative association with the unreliable person as children experienced only the reliable or the unreliable experimenter. Nevertheless, the studies we mentioned point to a level of cognitive engagement that goes beyond the unreflective responses to evidence typical of ordinary belief-formation not involving conscious evaluation of the evidence.^15^

In this section we have provided a prima facie philosophical and empirical case to consider the possibility that children are capable of a basic form of reflective thought before they are capable of articulating thoughts about other thoughts. This speaks in favour of the continuity between unreflective and reflective responsiveness to evidence. Let’s now consider two objections. The first, in § 4, questions the suggestion that one can acquire a defeater like < the source is unreliable > without having the capacity to formulate thoughts about other thoughts. The second, in § 5, tries to accommodate all that has been said so far within a reflectivist framework advocating dualism.

## A First Objection

Some might object to the claim that entertaining a thought like < the source of evidence is unreliable > does not require the ability to formulate thoughts about other thoughts along the following lines. Epistemic reasons are essentially relational: They are reasons *for* adopting or revising a doxastic attitude. If so, a thought like < the source of evidence is unreliable > in the example above is elliptical for < the source of the evidence for believing* p* is unreliable > , which is a thought with reference to another thought; namely, the (putative) belief that *p*. If this is right, only subjects who grasp the concept of belief and are already versed in reflective thinking can acquire < the source is unreliable > in the way outlined in the previous section, against the suggestion that the cognitive abilities needed to do so are already available at the unreflective level.

Someone sympathetic with this line of thought might account for the empirical studies summarized at the end of the previous section either in a deflationary manner by appealing to some unreflective sensitivity to the reliability of a source of evidence, or in an inflationary way by suggesting that the children in the studies mentioned above have at least an implicit grasp of the concept of belief. The first option would be in line with the common view that children reach the so-called “age of reason” around 6 years of age; the second option would make them reflective subjects much earlier than commonly assumed. What both options have in common is that they are hospitable to the separation of unreflective and reflective responsiveness to reasons advocated by dualism.

Let us grant that epistemic reasons are essentially relational, and that < the source of evidence is unreliable > does include implicit reference to some doxastic attitude towards a proposition allegedly supported by the relevant evidence. This, we acknowledge, means that a *full grasp* of < the source of evidence is unreliable > demands the capacity to formulate thoughts about other thoughts. The objection is thus well-taken. However, it ignores the dynamism inherent to the process of acquiring grasp of a concept or a thought *for the first time*. Yet, the primary focus of the present investigation lies in how a child can come to formulate a thought like < the source of evidence is unreliable > for the first time, and thereby acquire some grasp of the concepts involved, *which the child did not have before*. The objection must therefore be read as a request to explain how the considerations made about the acquisition of, and response to, < the source of evidence is unreliable > relate to attaining grasp of the concepts involved—in particular, grasp of the epistemic concept of evidence. This is a fair request for clarification.

Let us thus consider the process of grasping the concept of evidence. Grasp of most concepts is a cognitive achievement in which we can distinguish a stage where the grasp is entirely absent, one where the grasp is only partial, and eventually one that may be described as full grasp.^16^ Simplifying for present purposes, we can say that full grasp of a concept consists in the ability to articulate a rule for its application. The relevant rule needn’t provide infallible necessary and sufficient conditions; rather, it can just be a generalization based on previous experience and already existent knowledge which, if counterexamples are discovered, may be revised.^17^ For example, one can exhibit full grasp of the countable concept < glass > by explaining that glasses are containers from which people can drink and which do not have a handle. Similarly, grasp of the concept < evidence > may be exhibited by saying that evidence is what supports taking a doxastic attitude towards a proposition under investigation—e.g., it suggests that the relevant proposition is true.

**Fig. 1:**
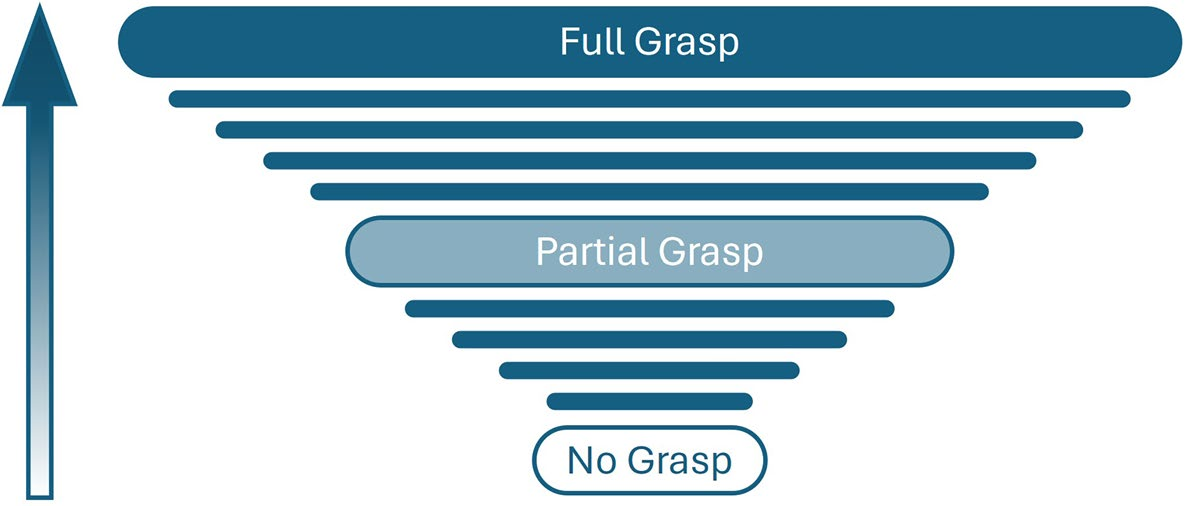
The stages of acquiring grasp of a new concept. Horizontal lines represent exposures to the objects, properties, or relations being conceptualized, which gradually accumulate to achieve partial and then full grasp of a concept

In both cases, full grasp is attained by going through a phase in which the grasp is only partial. A child might initially appreciate that liquids can be put in a glass, and only later understand that glasses’ main purpose is to enable people to drink. Perhaps the child has not yet realized that drinking is something different than eating, or she has not yet made the connection between seizing a glass with one’s hands and drinking (maybe she hasn’t seen that happening yet). Or she might still be unable to distinguish between cups and glasses. Still, at this stage the child can identify several glasses as belonging to the same category, thereby exhibiting a partial grasp of the concept < glass > . The cognitive abilities sustaining such partial grasp are *not* the result of understanding a rule or definition; rather, they are determined by perceptions, memories and dispositions that the child is not able to articulate in thought. Nevertheless, at this stage the child is already *using* the concept and we warrantedly ascribe to her thoughts like < a glass is on the table > or < the red glass is taller than the blue glass > based on her behaviour.^18^ Indeed, the child may well express such thoughts verbally. When the child’s ability to generalize in relevant circumstances is honed through practice and feedback, the child comes to formulate the thought < glasses are containers from which people can drink and which do not have a handle > . In doing so, she comes to understand a rule for the application of the concept and thereby acquires a full grasp of it. The practice and feedback that allow the child to acquire this full grasp can be thought of as repeated exposures to the objects, properties, or relations that the child is conceptualizing, each of which contributes to achieving full grasp. As shown in Fig. 1, it is the accumulation of such exposures—of which there may be many—that enhances grasp of the concept to the point that children can formulate a rule.^19^

Similarly, when a child is working her way towards full grasp of the concept < evidence > , she goes through a phase in which she responds to the normative force of the evidence without yet grasping a rule for the application of the concept. This occurs first at the level of unreflective responses in which the child responds to positive evidence and overriding defeaters. At this level, the child does not possess any grasp of the concept of evidence, she does not formulate any thoughts involving it, and she does not take any explicit evaluative perspective on her belief-formation and revision. Though there is initially no grasp of the concept of evidence, each unreflective response to evidence will contribute to acquiring some initial grasp of it, as shown in Fig. 1. Later, when the child makes a generalization like the one described in the previous section for the first time and as a result identifies a piece of evidence as likely to be misleading, she takes an explicit evaluative perspective on her (putative) evidence. In doing so, she begins to develop some grasp of the concept of evidence, and formulates a thought along the lines of < the source of evidence is unreliable > , even if she’s not yet able to articulate a thought expressing what the evidence is *for* (e.g., < the source of the evidence for believing *p* is unreliable >). At this stage, the child positively understands two main things. First, she appreciates that different pieces of evidence have something in common, even if she doesn’t understand yet what that thing is—i.e., all pieces of evidence provide support for some doxastic attitude. Second, she also understands that a subset of those pieces of evidence—those coming from the unreliable source, deserve a negative evaluation, which we express by a thought like < the source is unreliable > . At this stage, the child is yet unable to articulate these two pieces of knowledge into words, which would require full grasp of the concept of evidence. If so, the child can acquire and respond to undermining defeaters in the way described even if she is not yet in the position to understand that evidence concerns doxastic attitudes like belief, and can’t yet formulate thoughts about other thoughts. It is at junctures like this that reflective responsiveness to reasons begins.^20^

Considering a further objection in the next section will enable us to articulate in more detail the main cognitive mechanism at work in the transition from no-grasp to partial grasp and eventually full grasp of the concept of evidence: The acquisition of, and response to, a piece of higher-order evidence like < the source is unreliable > . Doing so will help to appreciate the fundamental role of unreflective responsiveness to evidence for rational agency.

## A Second Objection

Another retort that may be attractive to the advocate of dualism of reflective and unreflective responsiveness to evidence is the following. The status of genuine (i.e., reflective) rational subject is achieved by going through stages of cognitive development like those outlined, in such a way that when the process is completed the subject’s mind is transformed and it becomes something altogether different from her previous unreflective mind. Children who have the partial grasp of the concept < evidence > described in the previous section are children who are being transformed into rational subjects, and that transformation will be completed only when they will be able to articulate thoughts about other thoughts like < I believe that *p* because of *q* > . In short, the worry is that while we may have outlined some of the stages that lead to the transformation into rational subjects, we have not offered yet any reason to think that rationality is already at play at the unreflective level.

This concern must be handled with care. No one disputes that children’s cognitive abilities change over their development, and the label “transformation” may well be appropriate in some conversational contexts. For example, a 7-year-old who has become capable of full-fledged reflection can launch inquiries that she could not engage in as a 3-year-old, such as wondering what job she might want to have when she grows up. Thus, there is a sense in which the cognitive abilities have been transformed in the transition to reflective subject. But, by the same token we could say that the cognitive abilities of an 18-year-old who is well versed in integral calculus have undergone a transformation since, as a 7-year-old, she could barely master elementary arithmetic operations. So, in part, the issue concerns what is meant by “transformation.”

The advocate of the reflectivist approach holds that reflective responsiveness to reasons is inherently different from unreflective responses. She will promptly concede that, at the physiological and neurological level, there is no transformation, but only growth and expansion of a very plastic system, in particular in the pre-frontal cortex. So, the claim that the reflective mind is a different kind of mind pertains to a mentalistic level that is not reducible to the neurological activity of the brain. It pertains to the level of first-person normative thought where subjects, in the light of reasons or evidence available to them, consciously choose to do one thing rather than another or just form a belief rather than another. But, unless one is prepared to endorse a strong dualism whereby mind and body are entirely separate substances (as opposed to a more plausible predicate or property dualism), one must postulate some cognitive mechanisms linking the purely cognitive dimension of what happens in the brain and the dimension of normative thought.^21^ Thus, the reflectivist who isn’t willing to embrace old-fashioned mind–body dualism is committed to the claim that reflective responses to reasons are underwritten by cognitive mechanisms unavailable to unreflective subjects. If so, the reflectivist position may be refuted by showing that reflection is underwritten by mechanisms already available to unreflective subjects.

In effect, we have already illustrated an instantiation of such mechanisms in § 3: They are responses to overriding defeaters and generalizations. But to appreciate how the reflective part is grounded in the unreflective one, it will help to think of responses to evidence or reasons as functions taking some content as argument and delivering a doxastic attitude as value. Simplifying, when first-order evidence (e.g., perceptual content that it’s raining) constitutes the argument, the value will be a first-order belief directly supported by the evidence (e.g., < it is raining >). In this case responding to evidence works as a simple one-to-one function. But in the acquisition of the undermining defeater illustrated in § 3,^22^ responding to higher-order evidence works as a many-to-one function. The arguments are the negative beliefs formed in response to the overriding defeaters and the value is a belief expressing an assessment of the source that generated the misleading first-order evidence: < the source is unreliable > . So, in the case at hand, the acquisition of higher-order evidence is the result of a recursive procedure: A procedure that is applied to the result of a previous application of the same procedure; namely, responding to evidence. First, one acquires some pieces of first-order evidence (arguments of the function) and goes on to form the related first-order negative beliefs (values of the function); later, one takes those negated first-order beliefs as evidence (the new arguments of the function) and forms a higher-order belief (new value). In all this, there is no need to formulate any thought about other thoughts. The child who acquires the piece of higher-order evidence < the source is unreliable > in the way described in § 3 thereby reiterates the procedure of evidence-identification on its original results. First the child identifies the first-order evidence, and then uses it to identify the higher-order evidence. Such identification of higher-order evidence employs the same sensitivity to the epistemic features of one’s predicament that are involved in singling out appropriately first-order evidence in unreflective responses to reasons. However, that sensitivity has now developed a partial grasp of the concept of evidence, which allows the child to group together various pieces of evidence as belonging to the same category and to consciously evaluate them as members of that category. That is a basic form of reflective thought: The child looks at the evidence *as evidence* even if she does not fully understand yet what evidence is—in particular, she cannot articulate what evidence is evidence for; namely, a doxastic attitude.

The suggestion that a recursive procedure drives the identification of higher-order evidence is important for two reasons which, taken together, show how responding to undermining defeaters like < the source is unreliable > is a form of identifying and assessing evidence which is grounded in unreflective responses to evidence and yet it leads to the full-fledged reflection of thoughts about other thoughts.

First, it helps to see how one may ascend to the level of identification and assessment of reasons purely on the basis of the exercise of unreflective abilities. The identification of higher-order evidence requires one to single out another piece of evidence *as evidence* and yet, at bottom, it is a reiteration of the procedure of identifying first-order evidence on the result of that procedure. Young children who have become proficient in identifying and responding to first-order evidence are in a position to acquire and respond to higher-order evidence because the latter is, at bottom, just a recursive application of the former. Of course, the sort of recursive thought outlined demands cognitive alertness, computational power, memory resources and some initial conceptual sophistication. So, achieving it is not a trivial and automatic matter. Hence, the ascent to the level of the conscious identification and assessment of reasons is a real accomplishment. But its origin does not lie in some top-down reflective power. Rather, it’s normatively rooted in, and emerges naturally from, what subjects do at the unreflective level.

The second reason why the recursive engine at work in the identification of higher-order evidence matters is that it mirrors the recursion of formulating thoughts about other thoughts. It is thus well positioned to serve as a stepping stone towards the acquisition of the capacities that are constitutive of full-fledged reflective thought. The identification of higher-order evidence thus meets the directionality constraint for cognition that has a meta-dimension while falling short of thoughts about other thoughts, which was outlined by Perner (2012, p. 110). To move from responding to higher-order reasons in the illustrated naïve sense (which does not involve understanding what reasons are *for*) to engaging in reflective thought of the form < I [refuse to] believe that *p* because of *q* > , a subject will have to appreciate that thoughts and beliefs themselves can be reasons. One possible way in which this may be accomplished is by learning to deal with peer-disagreement, which in standard cases involves an inference from the contrasting opinion of a peer to the revision of one’s doxastic attitude.

Once one can identify and assess evidence as coming from a specific source, one is in a position to appreciate that there are various sources of evidence and that—in virtue of forming representations of the world in response to environmental cues like oneself does—peers and other subjects can be sources of evidence too. This additional step may be taken via a further generalization on what counts as a source. In noticing an analogy between the way in which others and oneself interact with the environment, one may begin to understand that subjects (including oneself) represent the world. That is the beginning of one’s grasp of the concept of belief, which will contribute to achieving a full grasp of the concept of evidence. Once one has begun to grasp that subjects represent the world, one is in a position to appreciate that another’s representation may differ from one’s own, and to treat them as evidence relevant to answer questions that one is interested in. At this point one begins to understand what evidence is evidence *for*: Doxastic attitudes or, in the beginning, some other form of representation or mere information-registration.^23^

While this is not the place to investigate in detail the mechanisms of the further transition to formulating thoughts about other thoughts, we can see how the capacity to respond to undermining defeaters is an important connection in the transition (*not* transformation!) from unreflective to reflective rational agency.

## Conclusion

We have argued that children’s ascent to the level of reflective responses to evidence can be driven by the recursive mechanism of the acquisition of the higher-order reason that a given source is unreliable, based on a generalization from overriding defeaters (first-order reasons) which affected beliefs formed following the indications of the source. The higher-order reason in question involves reference to other thoughts, but young children can’t fully articulate that yet. We have framed the point by saying that the child has a partial grasp of the concept of evidence, which falls short of grasping a rule for its application like < evidence is what supports taking a doxastic attitude towards a proposition under investigation > .

The proposal emphasizes that when one has a partial grasp of a concept, one is already engaging with it. Full grasp of a concept does not appear out of the blue; it builds on the intellectual and cognitive effort made with the resources available to the subject. In the case at hand, the most salient resources initially available are those of unreflective responsiveness to evidence and the capacity to generalize. The picture that emerges is one where full grasp of epistemic concepts arises from a partial grasp of them, which in turn begins with unreflective responsiveness to instantiations of them. In responding to evidence unreflectively, one has already begun one’s journey towards grasp of the concept < evidence > .

The possibility of acquiring and responding to some higher-order evidence on the basis of first-order thinking suggests that the transition from unreflective to reflective responsiveness to evidence is a natural progression of the development of one’s proficiency as a belief-manager. Rational-belief management is an effort to improve the coherence and accuracy of one’s set of beliefs: Any subject who engages in it aims to get rid of false beliefs and to replace them with true ones. This is key to improve one’s epistemic performances, even when one’s epistemic activity is confined entirely at the first-order, and it is essential to achieving one’s practical goals. Doing so requires sensitivity to overriding defeaters. Such sensitivity primes the alert and cognitively capable subject to notice underlying patterns of unreliability in sources which tend to deliver false or misleading information. The subject thereby *acquires* undermining defeaters such as < the source is unreliable > , *responding* to which involves looking at new evidence coming from the specific source not just for what it literally represents (e.g., the informant’s presence at a meeting), but also as having an illusory justificatory force (i.e., the information is deemed misleading). It is in virtue of the conscious appreciation of the mere illusory nature of their justificatory force that the evidence—now judged to be merely putative—is rejected.

Reflective responsiveness to reasons is thus something that emerges naturally from one’s engagement in unreflective responses. Nothing fundamentally new is at work in reflective thinking that wasn’t already operative at the unreflective level. The roots of reflective epistemic rationality—indeed, the roots of epistemic rationality *tout court*—lie in unreflective thinking. This is consistent with the acknowledgement that, in transitioning to reflective responsiveness to reasons one’s cognitive abilities undergo substantial change, which allows for entirely new investigations. It’s just that it’s driven by, and normatively grounded in, *what’s already there*.

If that is right, a dualistic view according to which the capacity for unreflective and reflective responses to evidence or reasons are separate is mistaken. Similarly, it would be a mistake to suggest that achieving the former capacity transforms one’s mind in a way that makes it different in kind from unreflective minds. Unreflective subjects, to whichever age and species they may belong, engage in the same activity of responding to evidence and managing and revising their belief-systems as reflective subjects do.

## Data Availability

Data sharing is not applicable to this article as no new data were created or analysed in this study.

## Footnotes

1 The clause that a reflectively justified belief can be *ex-post* sustained by conscious identification and evaluation of the evidence is there to acknowledge that beliefs initially formed automatically (e.g., basic perceptual beliefs) may still fall within the remit of the reflectivist view—provided that the relevant subject is capable of reflection.

2 Appeals to reflection as necessary to account for epistemic justification are common in advocates of access-internalism, according to which epistemic justification demands that justificatory factors are reflectively accessible (e.g., Bonjour, 1980; Chisholm, 1988; Ginet, 1975; Smithies, 2015; Steup, 1999). Some philosophers who are critical of access-internalism (e.g. Alston 1988 and Kornblith 2012) still use the term “reflection” primarily to talk about thinking about reasons or evidence. In more general discussions of rationality, appeals to reflection on reasons as essential to account for rational belief are to be found in McDowell’s (1994, e.g., p. 40) discussion of the “obligation to reflect on the credentials of the putatively rational linkages that one takes to govern the business of adjusting one’s world-view in response to experience,” Korsgaard’s (1996, e.g., p. 93) discussion of the authority of reflection whereby “the reflective mind cannot settle for perception and desire…[i]t needs a reason”, Boyle’s (2018, p. 112) suggestion that rational animals are those whose “thoughts and actions can be guided by an assessment of reasons, and [who] can adjust their beliefs and actions by reflecting critically on such assessments,” and in many other places. For a recent discussion of the notion of reflective responsiveness to evidence, see Melis and Monsó (2024, Section IV). Thanks to a reviewer for urging us to add these references.

3 In the Dual Process framework, reflective thought depends on working memory not just to present a problem, but also to allow the subject to find a solution via a series of stages. See Nagel (2014, § II) for more discussion.

4 We are simplifying, of course. Some question the potential of such tests to tell apart the intended notion of reflection from other more general competences (such as, reading and mathematical skills), and some have proposed fixes, such as think-aloud protocols.

5 Of course, being adults, the subjects who participate in cognitive reflection tests do have the ability to ask themselves whether the conclusion is well supported by the premises and whether the premises are justified. But inferential reasoning is available to subjects unable to entertain thoughts with epistemic concepts (i.e., unreflective subjects, in the sense that matters for us). For just one recent example of the vast literature discussing inferential reasoning in presumed unreflective subjects, see Call (2006).

6 See Kalpokas (2024) for a recent detailed discussion of the idea that perception comes with conceptual content.

7 For versions of this view, which is articulated differently by different proponents, see, e.g., Boyle (2012, 2016, 2018, 2024), Brandom (2000), Korsgaard (2009, 2018), Marcus (2021, 2022), and McDowell (1994, 2013).

8 Merely taking a dualist stance on reason-responsiveness is in principle compatible with mind–body dualism too but, for reasons briefly rehearsed in footnote 21 below, reflectivists are unlikely to be attracted to it.

9 A reviewer invites us to clarify the relevance of views that defend immediate perceptual justification based on seemings, such as Huemer (2001) or Pryor (2004). Such defences of immediate justification have emerged in the debate on scepticism and oppose views that demand that perceptual justification rests also on some additional warrant to discount the hypothesis that one may be in a sceptical scenario (e.g., Wright, 2004). Now, to the extent that the putative victim of sceptical scenarios is an adult human, immediate justification in this context applies primarily to reflective subjects. So, while immediate perceptual justification is clearly not inferential, it is not thereby a case of unreflective responses to evidence. To appreciate this, remember that the reflectivist view at the centre of the present investigation *does not* demand that subjects actually *base* their beliefs *on* the assessment of relevant evidence, but only that they are in the position to evaluate relevant evidence *after* the belief is formed. The debate on whether there is immediate perceptual justification is orthogonal to the question whether one can be justified in holding a belief without being in the position to identify the evidence in its support. One can embrace both immediate justification and the reflectivist view we discuss by demanding that one’s reasons for belief must be accessible on reflection. This is, roughly, the view defended by Bonjour in BonJour and Sosa (2003, e.g. p. 73). But one can also embrace immediate justification while dropping the requirement of the accessibility of evidence on reflection and thereby embrace unreflective justification in the sense that is relevant here. This is a view closer to Huemer’s (2001, e.g. pp. 177–8). In short, while some versions of seemings-based perceptual justification are hospitable to unreflective justification, they are generally silent on the issue of the relation between such unreflective justification and its reflective counterpart. They say even less on the developmental question that guides this paper; namely, how can subjects who are only capable of unreflective responses to evidence acquire the capacity to reflect on their reasons for belief? Another terminological clarification may be helpful here. Some philosophers, following Austin (1962, p. 115), deny that basic perceptual beliefs are evidence-based. According to these philosophers, one can only have evidence in cases where the question whether *p* is, in some sense, open to investigation but, so the story goes, perceiving that *p* does not leave room for wondering whether *p*. Yet, these philosophers must still allow for some basis for those beliefs—a basis that one would invoke if the justification of those beliefs is challenged. For example, if one’s perceptual belief that *p* is challenged by questions like “how do you know that *p*?” or “what are your reasons to believe *p*?”, it’s perfectly natural to respond by saying “I saw that *p*.” We use the term “evidence” or “reasons” in a wide enough sense to include such cases.

10 See, e.g., Christensen (2010) and Lasonen-Aarnio (2014) for discussion of some well-known examples, and Whiting (2020) and Horowitz (2022) for overviews of the debate on higher-order evidence. It may be worth noting that in the literature the term “higher-order evidence” is often used as virtually synonymous with “higher-order *defeating* evidence.” This is largely because higher-order defeating evidence makes some philosophical puzzles perspicuous in a way that higher-order *confirming* evidence does not and it is thus more widely discussed. We will discuss primarily defeating higher-order evidence too, but defeating and conforming higher-order evidence are two sides of the same phenomenon and, following Whiting (2020, p. 789), it is more accurate to introduce the notion of higher-order evidence in a way that is neutral between the two.

11 Rohwer et al. (2012) conducted a hiding task aimed at testing children’s degree of awareness about their own epistemic states in which, among other things, 3- to 7-years-old were asked to justify their correct claims of knowledge or lack thereof. Children under 6 years of age had considerable difficulties to justify claims of ignorance (21% correct), and children under 5-years of age struggled to justify claims of knowledge (23.4% correct). By contrast, 6- and 7-year-olds by and large provided appropriate replies in support of ignorance (81.3% correct), while 5- to 7-year-olds were largely able to justify their knowledge (85% correct). In the screen task run by Butler et al. (2020), 3- to 7-year-olds had to: (i) select a generally reliable informant over an unreliable one; (ii) choose between informants who verified their information and those who did not in specific circumstances (regardless of their general reliability); (iii) provide justification for their choice in (ii). While children of all ages selected the reliable informant above chance (task i), 4- and 5-year-olds were above chance in choosing the informant who verified the information (task ii), 6- and 7-year-olds were able to provide more pertinent justifications for their choices than 4- and 5-year-olds (task iii). In a selective information seeking study run by Blakey et al. (2021), 3- to 8-year-old children were asked to explain how they selected the information needed to complete the task. Only from 5 years old were a substantial proportion of children able to offer a *pertinent*, if not always correct, reply (about 50% in 5-year-olds, 60% in 6-year-olds, 75–80% in 7-year-olds, and over 90% in 8-year-olds). The proportion of *correct* replies among 5-year-olds was about 15%, 30% in 6-year-olds and 50% in 7-year-olds.

12 Children who answered both explicit judgement questions correctly chose to endorse the reliable informant in 67% of the novel test trials (Koenig et al., 2004).

13 We can’t expand on this here but, roughly, to determine whether children have responded to an undermining defeater like < the source is unreliable > there needs to be a change of context within the experiment. The change of context allows us to assess whether children have assessed the evidence coming from the source as being misleading and can generalize this to other types of evidence provided by the same source. See Blakey et al. (2024).

14 The delay of gratification task in which children’s responses to the experimenters were compared was a different context to the circumstances in which the children learned about their reliability/unreliability.

15 *Mere* unreflective belief-revision—the belief-revision of unreflective subjects—involves at least the following cognitive abilities: (i) the capacity to grasp concepts representing salient features of their environment needed to form relevant first-order beliefs; (ii) the capacity to make simple inferences, such as generalizations like < dogs bark > based on the observation of some dogs who barked, (iii) a capacity to monitor their own cognitive endeavors manifested in decisions about when to start or stop specific inquiries in relation to how important the inquiry is and how it is progressing. None of these feats requires any familiarity with epistemic concepts, and thus needn’t involve performing conscious epistemic evaluations, in the relevant sense. For discussion of concept-possession in unreflective subjects, see, e.g., Rowlands (2012, Chapter 2), and Carruthers (2009). For generalizations in young children, see Kimura and Gopnik (2019), even if they infelicitously (for present purposes) use the term “higher-order belief” to refer to the relevant generalizations. For an illustration of cognitive monitoring without conscious epistemic evaluations, see Carruthers (2008).

16 Concepts are typically acquired through experience in the sense that their acquisition is triggered by perceptual stimulation or interaction with others. This is true also of concepts (e.g., < red > , < round >) that in the developmental literature are commonly described as innate, as their acquisition is the result of normal human maturation rather than learned through immersion in one culture or dependent on a specific range of experiences. To say that a concept is innate in this sense does not entail that the concept is present at birth. Cf., e.g., Carey (2009, p. 12), Burge (2007a, b, p. 168) and Laurence and Margolis (2024, pp. 35–36). We say that “most” concepts are acquired through a process in which stages can be distinguished because, according to a common assumption that Laurence and Margolis (178ff) dub “Acquisition by Composition” (ABC), *primitive* concepts—i.e., concepts that cannot be assembled from simpler concepts—cannot be learned. The ABC model of concept development has it that all new concepts are assembled from more basic constituents and thereby requires some set of unlearned primitive concepts (to be specified). However, the idea that primitive concepts cannot be learned is disputed. For example, Laurence and Margolis describe several mechanisms through which primitive concepts are learned, such as abstraction (156ff) and what they call “syndrome-based acquisition systems” (562ff). Similarly, Carey (2009, Chapter 8) proposes that the concepts of positive integers are acquired through a rather demanding process of “conceptual bootstrapping”. We don’t need to take a stand on the debate on the learnability of primitive concepts. All that we are committed to is that some concepts are learned, and that < glass > and < evidence > are instances of such concepts. Our proposal is inconsistent with Fodor’s (2008) radical concept nativism, according to which no concept, be it primitive or complex, can be learned. We are happy to bite this bullet, and we refer to Laurence and Margolis (pp. 533–600) for criticism and extensive discussion on the significance of Fodor’s arguments.

17 We take this notion of full grasp of a concept to be neutral with respect to the various views on the nature of concepts in terms of, e.g., mental representations, abilities, and abstract objects.

18 See Bloom (1996) and Matan and Carey (2001) for empirical evidence that artifacts concepts like < glass > are learned and that the function of the relevant artifact is central to such learning. Note also that partial grasp of concepts is a phenomenon not limited to children. The phenomenon underlies famous thought experiments, such as “twin-earth” and “arthritis,” that have led many philosophers to endorse externalism about mental content. For discussion of incomplete understanding in adults see Burge (2007b, pp. 174–179).

19 The picture just outlined is compatible with virtually all accounts of conceptual development, which describe grasp of *new* concepts as a process in which different phases can be distinguished. For example, Laurence and Margolis (2024, pp. 551–552) distinguish between the phases of “exploratory search” (roughly corresponding to our partial grasp), “concept formulation” (roughly corresponding to our full grasp) and an additional “final verification” phase where the subject goes on to further test the rule for the application of the concept she formulated.

20 Our suggestion that the categorization of objects falling under a concept—which is driven by perceptions, memories and dispositions—is part of a process that leads to the articulation of a rule for the application of the concept is supported by empirical studies showing that concepts operate on both ends of the spectrum running from automatic and low-effort system 1 thought to deliberate and effortful system 2. See, for example, Ashby and Valentin (2017) and Gabay et al. (2023) for evidence that concepts can be learned either in implicit and automatic manner or through rule-based deliberate reasoning. This is suggestive of precisely the sort of continuity that advocates of Dual Processes and Dual Factor views recalled in Sect. 2 advocate at a *descriptive* level. Our suggestion that, by learning to respond to undermining defeaters, one begins the process to acquire the concept of evidence makes a case for the same sort of continuity at the *normative* level of substantive rationality.

21 We take it that contemporary reflectivists are not attracted to traditional “substance” mind–body dualism which, among other things, struggles to account for the interactions between mind and body, and appears to have been empirically disproved by famous brain-damage cases such as those involving Phineas Gage (Damasio, 1994). Rather, they would endorse either the mind-mind dualism outlined in § 2 above, or a version of mind–body dualism that allows that individual thoughts are instantiated in the brain. Examples of such mind–body dualism are “predicate” and “property” dualism, both of which allow for token-identity between each thought and some neurological activity while denying type-identity between thoughts as a kind and some kind of neurological structure or activity. Loci classici for predicate and property dualism are Davidson (2001) and Nagel (1974) respectively.

22 For convenience, here is a schematic reconstruction. First, the subject S is misled several times by the target source: that is to say, S acquires several *overriding* defeaters affecting beliefs < *p* > , < *q* > , < *r* > … formed following the indications of the source. Those beliefs are thereby revised to < not-*p* > , < not-*q* > , < not-*r* > … Second, the subject notices that several beliefs formed following the indications coming from that specific source have been revised and makes the generalization that the source in question is unreliable. The subject has thereby acquired an undermining defeater.

23 See Burge (2018) for the view that very young children and non-human animals ascribe to others informational states that fall short of belief and mental representation.
